# Mr. Bartlett, on Nicotiana

**Published:** 1804-11-01

**Authors:** Mich. Bartlett

**Affiliations:** Member of the Royal College of Surgeons, and Apothecary to the Finsbury Dispensary. St. John's Square


### *To the Editors of the Medical and Phyfical Journal.*

GENTLEMEN,

I CONCEIVE that your very valuable and esteemed Journal must be highly important to the medical world, inasmuch as it affords a ready and easy channel for the conveyance of medical truths and practical observations; a few plain and well authenticated facts being of more real utility in the practice of physic, than whole volumes of theoretical disquisitions; and I consider it an obligation incumbent upon every member of the profession, to contribute, as far as it may lay in his power, towards the promotion of medical science; for in a subject buried under so much obscurity as that of the animal economy, it is not to be wondered at, that the practitioner should be often embarrassed in his prescription, and obliged to regulate his conduct by conjecture or uncertain analogy. Impressed with the importance of this idea, I shall beg leave to state what has been the result of my experience with regard to the operation of a remedy, which, although generally known, does not seem to have had that attention paid to it which its merits appear to me to demand.

I am firmly persuaded, that we have not in the whole Materia Medica a more useful or powerful agent than *nicotiana*, when properly administered, in cases of strangulated hernia, or obstinate constipations of the bowels; as, for the most part, they arise from spasmodic stricture. I do not know a remedy that is equally efficacious, its almost immediate effect being that of inducing a state of general relaxation; a state the most favourable that can possibly be conceived for the relief of such complaints.

The principal objection which I understand to have been raised against its more frequent use, is the alarming state which its operation often induces; but it should be recollected, that the patient's life is in a much more perilous state previous to its exhibition, for it is only to be employed when all the more lenient remedies have failed in producing the desired effect.* I have applied it myself,

* Nicotiana, although so valuable a medicine, ought to be employed with great ca e and discrimination; Mr. Astley Cooper has stated two instances in which it has proved fatal. The late publication of this eminent surgeon on the subject of hernia will be read by every student who has a due regard to his professional improvement.

and have seen it applied, in a variety of cases, and do not know a single instance of its failure. In two cases, which have fallen under my own particular observation, the success of its application will appear to have been of the most decisive nature. The cases, as they apeared in succession, I shall now take the liberty to lay before you.

Mary Brett, a woman of a naturally healthy and vigorous constitution, became a patient of mine, labouring under symptoms of strangulated hernia. Upon examination, I found that she had inguinal hernia on both sides; in that on the left, the intestine was evidently strangulated; she had very severe pain in the part, attended with nausea and vomiting. She had not had any evacuation downwards for three days previously to my seeing her. I attempted to reduce the hernia by the usual mode of practice, such as placing the patient in a supine posture with her knees elevated, and brought nearly in contact, to relax the facia of the thigh; then applying gradual and moderate pressure to the tumour, but without effect; and, indeed, the operation could not be persevered in, the pain it occasioned was so intolerable that she would not suffer me to persist. I ordered for her the following pills, ℞. Ext. colocynth. comp. ʒß. calomel pt. gr. xij. opii gr. j. M. fiat pil. vj. cap. ij. omni. 2nd. hora donec alvus respond. In the intermediate time, oily enemas were directed to be thrown up, and the hot bath to be made use of.

It was in the afternoon that I saw her. I called again on the following morning, to see what effect these remedies had produced, when I found the symptoms rather aggravated than appeased; the pills were retained for about an hour, and then ejected from the stomach; the pain in the tumour continued with unabated violence, attended with a spasmodic constriction of the abdomen; and there was evidently an inversion of the peristaltic motion of the intestine, a quantity of stercoraceous matter being contained in the vessel in which she had discharged the contents of her stomach. Conceiving now that no time was to be lost, I mentiond to her friends the necessity of performing an operation, as the only probable chance of preserving her life; but this they strongly objected to, urging that, at her advanced period of life, little advantage could be derived from an operation; and, indeed, it could not be insisted upon with any degree of confidence on the part of the practitioner, but might rather be considered as the forlorn hope. *Nicotiana* now became the dernier resort, of of which I ordered a drachm of what is vulgarly called shag tobacco, to be infused in a pint of boiling water, and two-thirds of this infusion to be injected immediately; the remainder in half an hour, if the former should not have had the desired effect. Upon my next visit I was very much surprised to find my patient entirely relieved from her complaint; the enema had operated almost immediately after its application, the hernia was reduced, the pain had subsided, and nothing now remained but to support the patient's strength by cordial and tonic medicines.

Thomas Cause, a young man about twenty-three years of age, by trade a compositor in a printing-office, became a patient of mine for the cure of colica pictonum. His disease was characterized by the following symptoms: violent pain about the umbilical region, extending across to the right hypogastric region; nausea, and vomiting of bilious matter; body costive; pulse full, and rather turgid. In addition to these symptoms he had violent spasmodic contractions about the abdominal cavity; the pain which it gave rise to was so distressing, that he cried out like a woman in labour, and the parts became so sensible to the touch that he could not bear the slightest pressure, not even that of the bed clothes. ℥x of blood were taken from him, an emetic was administered, and an aperient mixture directed to be taken, with a view to evacuate the bowels. On my next visit I found that these remedies had not afforded any relief to the patient; the vomiting and pain continued as violent as ever, and no evacuation had been produced by the opening mixture. I now directed an oily enema, with thirty drops of tinct. opii, to be thrown up, and the warm bath to be made use of; I likewise prescribed for him six grains of calomel to be taken immediately. I saw him again on the next day, which was the fourth from the attack, when I found that the bowels continued obstinately constipated, no evacuation having been produced by any of the means made use of; the patient was evidently sinking from the constant action that had been kept up during this time; the spasms were so frequent and distressing, that he could get no rest. I now determined to try the *nicotiana*, in the same proportion as in the before mentioned case, when I am happy to say its effects were equally decisive and beneficial; its operation induced a state bordering upon syncope, and the evacuation which followed was so profuse that it became necessary to check it by opiates, and to support the patient by nourishing broths and cordial medicines. He continues well, and has not had any unpleasant symptoms since.

*(Query)* Might not this remedy be employed with good effect in cases of tetanus and trismus, as they depend upon spasmodic contractions of the muscular fibres?

I am, &c.

*St. John's Square, Sept.* 22, 1804.

MICH. BARTLETT,

Member of the Royal College of Surgeons, and Apothecary to the Finsbury Dispensary.

